# Natural Antisense Transcripts: Molecular Mechanisms and Implications in Breast Cancers

**DOI:** 10.3390/ijms19010123

**Published:** 2018-01-02

**Authors:** Guillaume Latgé, Christophe Poulet, Vincent Bours, Claire Josse, Guy Jerusalem

**Affiliations:** 1 Laboratory of Human Genetics, GIGA-Institute, University of Liège, 4500 Liège, Belgium; g.latge@doct.ulg.ac.be (G.L.); christophe.poulet@ulg.ac.be (C.P.); vbours@ulg.ac.be (V.B.); c.josse@chu.ulg.ac.be (C.J.); 2 Center of Genetics, University Hospital (CHU), 4500 Liège, Belgium; 3 Department of Medical Oncology, University Hospital (CHU), 4500 Liège, Belgium; 4 Laboratory of Medical Oncology, GIGA-Institute, University of Liège, 4500 Liège, Belgium

**Keywords:** non-coding RNA, lncRNA, natural antisense transcript, natural antisense transcripts, breast cancer, next generation sequencing, gene expression regulation

## Abstract

Natural antisense transcripts are RNA sequences that can be transcribed from both DNA strands at the same locus but in the opposite direction from the gene transcript. Because strand-specific high-throughput sequencing of the antisense transcriptome has only been available for less than a decade, many natural antisense transcripts were first described as long non-coding RNAs. Although the precise biological roles of natural antisense transcripts are not known yet, an increasing number of studies report their implication in gene expression regulation. Their expression levels are altered in many physiological and pathological conditions, including breast cancers. Among the potential clinical utilities of the natural antisense transcripts, the non-coding|coding transcript pairs are of high interest for treatment. Indeed, these pairs can be targeted by antisense oligonucleotides to specifically tune the expression of the coding-gene. Here, we describe the current knowledge about natural antisense transcripts, their varying molecular mechanisms as gene expression regulators, and their potential as prognostic or predictive biomarkers in breast cancers.

## 1. Introduction

After an international effort, the scientific community has revealed that up to 90% of the human genome is transcribed. Thanks to the FANTOM project (functional annotation of the mammalian genome, available online: http://fantom.gsc.riken.jp/), started in 2000 with the mouse genome [1,2], which was quickly followed by the human genome in 2003 by the ENCODE project (encyclopedia of DNA elements, available online: https://www.encodeproject.org/) [3,4], we know that 98% of the human genome is composed of non-coding (nc) sequences, previously considered “Junk DNA” due to their heterogeneity, low expression levels, and unknown functions [5,6,7,8,9,10,11]. This huge part of the transcriptome could therefore play a role in protein-coding (pc) RNA expression regulation. Databases specialized in genome annotation, such as the GENCODE project (encyclopædia of genes and gene variants, available online: http://www.gencodegenes.org/) [12,13], specialized in ncRNA, such as the NONCODE (integrated knowledge database dedicated to ncRNAs, especially lncRNAs, available online: http://www.noncode.org/) and RNAcentral projects (the non-coding RNA sequence database, available online: http://rnacentral.org/) [14,15], or specialized in human long non-coding RNAs (lncRNAs), such as the LNCipedia project (a comprehensive compendium of human long non-coding RNAs, available online: https://lncipedia.org/) [16], are now illustrating this new vision of the transcriptome.

**Figure 1:**
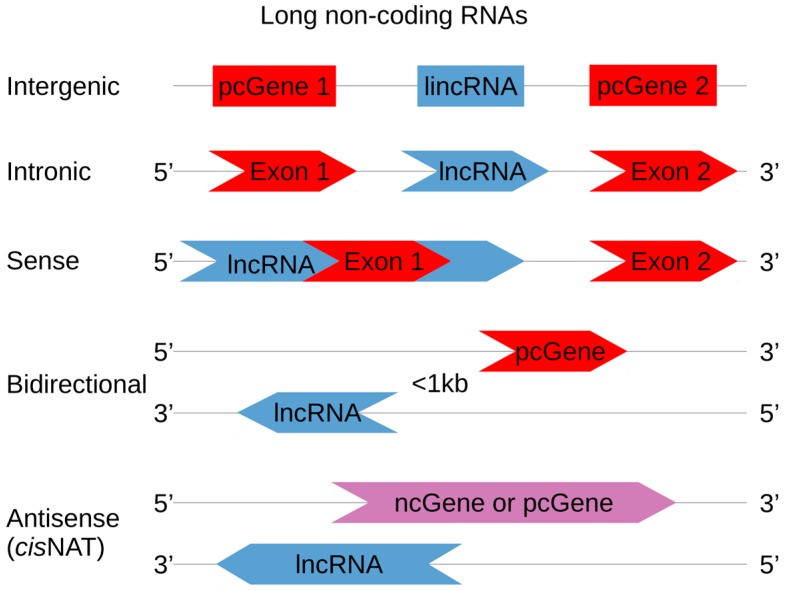
lncRNA classification according to their orientation and position in the genome. lincRNAs are located between two *pcGenes*, regardless of their orientation. Intronic lncRNAs are entirely encoded in *pcGene* introns, while sense lncRNAs overlap *pcGene* exons. Bidirectional lncRNA transcription starts less than 1 kb from a *pcGene* transcription start site and goes in its opposite direction. *Cis*-NATs (natural antisense transcript) are RNA sequences that are transcribed from the two strands of the same genomic locus, in the antisense direction. NAT pairs can be protein-coding sequences (pc, red colored) or non-coding sequences (nc, blue colored), forming nc|pc, nc|nc or pc|pc pairs. NAT pairs that are nc|pc or nc|nc sequences only belong to the lncRNA classification (purple colored sequences are pc or nc).

Non-coding RNAs are classified according to their transcript length. With few exceptions, a 200-nt arbitrary threshold is used to separate short and long ncRNAs [17]. The long non-coding RNAs (lncRNAs) make up the largest portion of ncRNAs. With approximatively 98% of the genome containing non-coding regions and only 1.1% containing exons, it is obvious that many lncRNAs do not overlap exons. lncRNAs are classified according to their original genomic location and their context when compared to a protein-coding gene (*pcGene*) [18,19,20]. Figure 1 depicts the five current categories of lncRNAs, named intergenic, intronic, bidirectional (or divergent), sense and antisense. Sense and antisense lncRNAs are defined according to the nearest *pcGene* position. Both can overlap, partially or entirely, one or more exons of a *pcGene* [2]. Bidirectional lncRNA transcription starts close to a *pcGene* (less than 1 kb from the *pcGene* transcription start site) and proceeds in the opposite direction as *pcGene* transcription. Intronic lncRNAs are transcribed entirely from introns, and do not overlap with any exon [20,21,22]. Those in intergenic regions are named long intergenic non-coding RNAs (lincRNAs), and their transcription can occur in both directions [23,24]. Some ncRNA classification exceptions are also worth noting. A few lncRNAs, such as BC1 or snaR, contain less than or close to 200 nt, but they are classified as lncRNAs in the lncRNAdb database (the reference database for functional long noncoding RNAs, available online: http://lncrnadb.org/) [25]. Other lncRNAs can reach lengths of 1 Mbase and are thus called very long intergenic RNA (vlincRNA) [26].

lncRNAs are defined as endogenous cellular RNAs without a significant ORF (open reading frame) [27,28,29]. However, some ncRNAs containing an ORF smaller than 100 amino-acids may be classified as lncRNAs [27]. The known biological roles of lncRNAs are very heterogeneous and cover various molecular and cellular functions such as *pcGene* regulation [30], stem cell pluripotency and differentiation [31], allelic expression [32], cell cycle control [33], apoptosis and senescence [34], heat shock response [35], and control of chromatin modifications [36]. It is worth noting that lncRNAs are found in all tissues and show pronounced tissue-specific expression. Their cellular location may vary, probably reflecting their function [20,37,38]. There is a structural similarity between lncRNAs and mRNAs, in the sense that they may be multi-exonic, 5′ capped, 3′ polyadenylated, and spliced [23]. RNA polymerase II (RNA Pol II) is responsible for the transcription of most of the lncRNAs, and their expression is under the control of promoters and enhancers, that can be induced by external stimuli [23].

## 2. Generic Definition of NATs

Natural antisense transcripts (NATs) are coding or non-coding RNA sequences that are complementary to and overlap with either protein-coding or non-coding transcripts [39]. As 98% of the transcriptome is non-coding, the vast majority of paired transcripts are composed of nc|nc or nc|pc pairs. Therefore, NATs are defined in regard to the relative genomic position from their paired transcript origins, in cis or in trans. *Cis*-NAT pairs are transcribed from the opposite strand of the same genomic locus and display perfect RNA|RNA sequence complementarity with the opposite strand transcript (if no RNA modifications, such as RNA editing, occur). *Trans*-NAT pairs are transcribed from different genomic loci, and the two RNA molecules may hybridize to each other with imperfect RNA|RNA sequence complementarity [40,41].

Because whole genome sequencing of the antisense transcription has only been available for less than a decade, many NATs were described as lncRNAs without information about the co-existence of other transcripts from the same genomic origin. This convergence between NAT and lncRNA classifications may thus lead to some confusion in the literature and will probably disappear with the increasing knowledge in the antisense transcription field.

## 3. NAT: Structure, Localization, and Expression Regulation

Like lncRNAs and mRNAs, NATs may be capped and poly-adenylated transcripts that are maturated to excise introns. NAT expression is also controlled by promoters and enhancers. In addition, many examples of bidirectional promoters that control transcript expression originating from both strands are described in the literature [42,43]. In this case, several transcription factors, such as GABPA or E2F1, are preferentially implicated [44,45,46]. NATs may originate from cryptic promoters that are then inserted within the intronic regions of a gene or close to the transcription start site of neighboring genes [43,47,48].

NATs accumulate preferentially in the nucleus, associating with chromatin, unlike coding mRNAs which accumulate in the cytoplasm. NATs are also found in other cellular compartments, such as mitochondria, and have been reported to accumulate at polysomes [3,18,49]. Moreover, NAT expression is closely linked with the activity of their sense or neighboring genes [43].

## 4. NAT: Role, Function and Mechanism of Action

The biological significance of NATs remains under scientific investigation with major key questions yet to be answered. Specific *pcGene* regulation by their corresponding overlapping ncNATs has been reported. Our team and others have shown that up to 50% of the *pcGenes* also express ncNATs [2,39,50] and that transcript levels of nc|pc pairs are often tightly correlated [39,46,50]. Altogether, this suggests that NATs could be implicated in a new level of gene expression regulation [5,51]. 

Both transcriptional and post-transcriptional regulations of expression have also been explained as the result of the creation of natural sense and antisense transcript pairs. The regulatory processes implicated can be more or less complex, ranging from simple transcriptional interference to modulation of chromatin changes or the formation of double-stranded RNA (dsRNA). The latter leads to RNA masking, RNA interference or RNA editing [52]. 

Several examples of *pcGene* expression regulation by their NATs are described hereafter to illustrate the different molecular mechanisms of action.

### 4.1. Action in Cis or Trans

**Figure 2:**
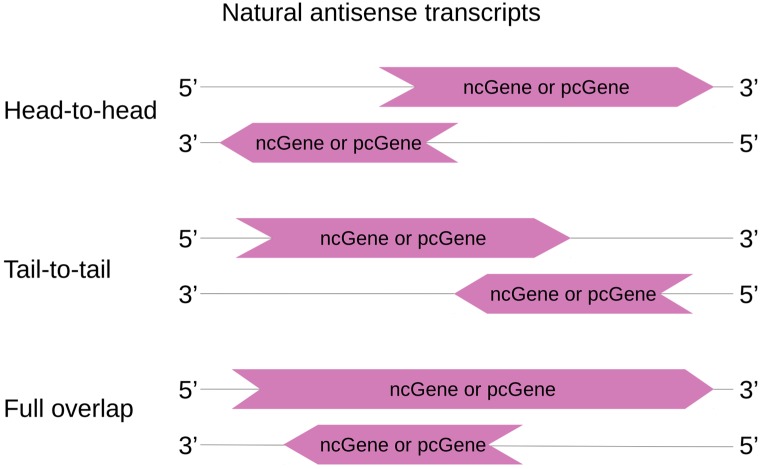
*cis*-NAT classification. cisNAT pairs can be protein coding sequences (pc) or non-coding sequences (nc), forming nc|pc, nc|nc or pc|pc pairs. In head-to-head orientation, sense and antisense transcripts overlap on their 5′ ends. Inversely, tail-to-tail describes an overlap of the 3′ ends. In a full overlap (or embedded overlap), one transcript is totally included in the other one.

While NATs are more likely to handle regulation of other genes in cis, they may also tune gene expression elsewhere in the genome by trans regulation. Based on the definition of overlapping genes from Makalowska et al., *cis*-NATs are here classified according to the relative position of the DNA coding sequence of the RNA transcripts [53]. Three categories can thus be described and are depicted in Figure 2: (1) “head-to-head”, where sense and antisense transcripts overlap on their 5′ ends; (2) “tail-to-tail”, where sense and antisense transcripts overlap on their 3′ ends; and (3) “embedded overlap” (also called “full overlap”), where one of the entire transcript overlaps the other. 

### 4.2. Transcriptional Interference

**Figure 3:**
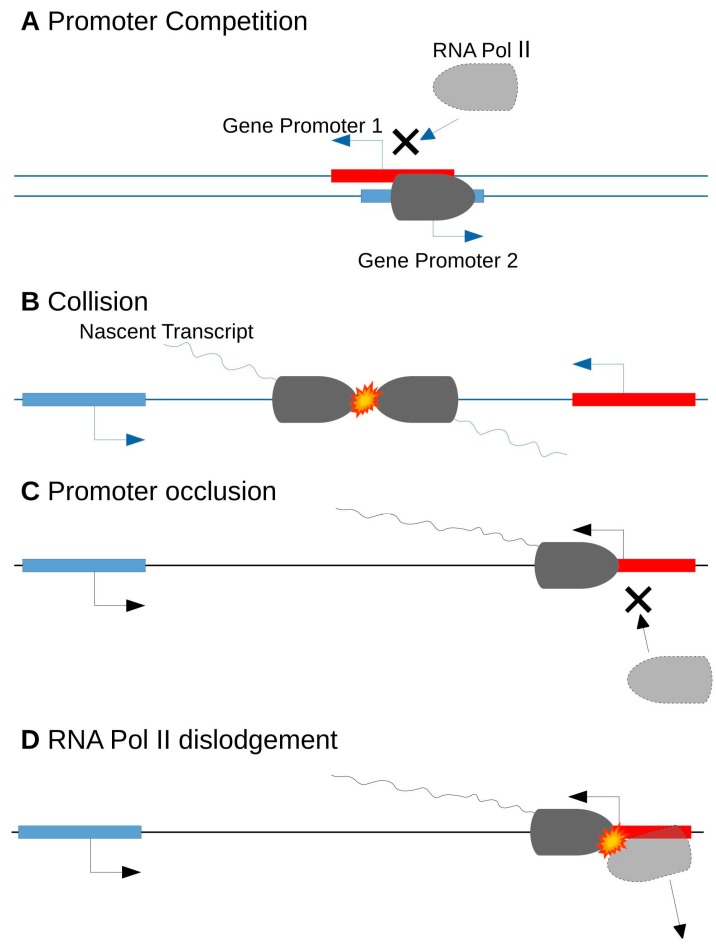
Transcriptional Interference: (**A**) in the initiation phase, promoters of head-to-head NATs are competing for the use of RNA Pol II and common regulatory elements; (**B**) in the elongation phase, interference can occur after the following events: a collision between RNA Pol II complexes, leading to a machinery blockage; (**C**) a promoter occlusion by RNA Pol II during the antisense transcript; and (**D**) a RNA Pol II dislodgement by the RNA Pol II standing on the opposite strand, when the first one was too slow to start. Promoters of protein coding sequences are represented in red, and promoters of non-coding sequences in blue. RNA pol II enzyme is represented in dark grey when able to transcribe the sequence, and light grey when its binding and thus activity, is prevented.

Antisense transcription can modulate in cis the sense transcription of the opposite strand, although this effect may not be caused by the pairing of the RNA molecules themselves. The proximity of the two transcriptional events, sense and antisense, leads to a downregulation of both transcripts [54]. Transcriptional interference can occur during the initiation or elongation phases of transcription. In the initiation phase, promoters of head-to-head NATs are competing for the use of RNA Pol II and common regulatory elements (Figure 3A). In the elongation phase, interference can occur after the following events: a collision between RNA Pol II complexes, leading to a machinery blockage (Figure 3B); a promoter occlusion by RNA Pol II during the antisense transcript elongation (Figure 3C); or an RNA Pol II dislodgement by the RNA Pol II standing on the opposite strand, when the first one was too slow to start (Figure 3D) [54]. It is worth noting that the transcriptional interference investigation field is still young and that formal proof of gene expression regulation by this mechanism was only recently reported [55]. Nevertheless, a negative correlation between sense and antisense transcript levels are less frequently observed than a positive correlation or no correlation. This suggests that only a minority of NATs could be involved in transcriptional interference processes [50,56,57,58,59].

Despite difficulties in discriminating transcription interference from gene expression regulation by RNA transcripts, Stojic et al. [55] have demonstrated such a mechanism by screening an siRNA library. Whereas nearly all siRNAs dampen GNG12-AS1 (a non-coding natural antisense transcript of the tumor suppressor coding gene DIRAS3) post-transcriptionally, siRNA targeting exon 1 of GNG12-AS1 downregulates its transcription by recruiting Argonaute 2 and inhibiting RNA polymerase II binding. In this case, the active transcription of GNG12-AS1 causes the transcriptional silencing of *DIRAS3*, leading to increased cell proliferation. 

### 4.3. Chromatin Modification

**Figure 4:**
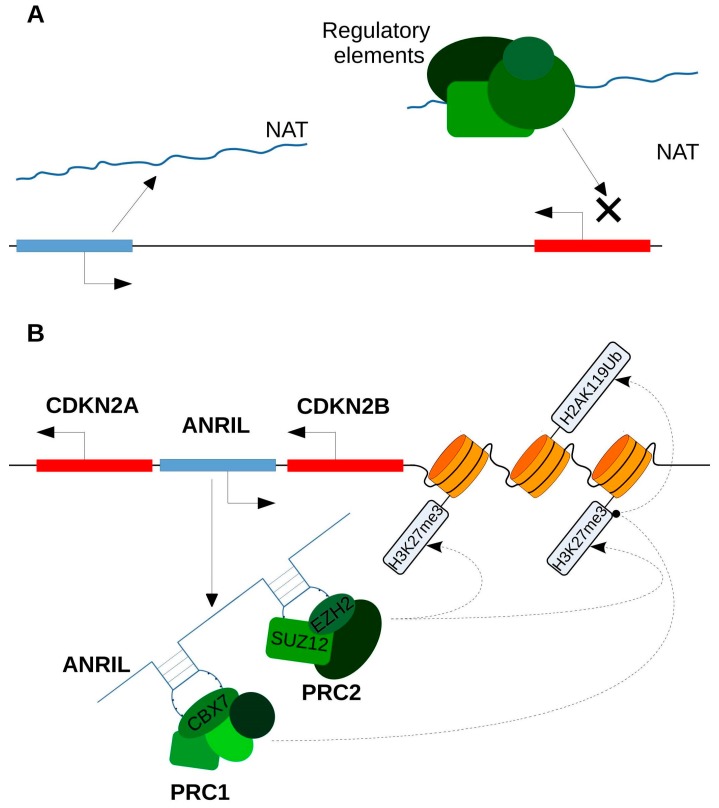
ncNATs (non-coding natural antisense transcripts) may regulate the expression levels of the sense pcGenes (protein coding genes) by regulating chromatin modifications by the following. (**A**) A decoy mechanism: The NAT binds a protein complex that can trigger chromatin modifications and prevents, by competition, this complex from binding the sense transcript. This complex can also prevent the interaction of the sense gene with RNA Pol II (RNA polymerase II); (**B**) a tethering mechanism, such as ANRIL (antisense non-coding RNA in the INK4 locus): ANRIL recruits PRC2 (polycomb repressive complex) through interaction with SUZ12 (suppressor of zeste 12 homolog) and EZH2 (enhancer of zeste 2 polycomb repressive complex 2 subunit) components and PRC1 by binding CBX7 (chromobox homolog 7). Next, PRC2 silences the INK4 locus expression by inducing H3K27 tri-methylation, and PRC1 maintains a repressive chromatin structure by mono-ubiquitination of H2AK119. Protein coding sequences or promoters are represented in red, and non-coding in blue.

ncNATs may regulate the expression levels of the sense *pcGenes* by regulating chromatin modifications. Such epigenetic modifications encompass DNA methylations of cytosine in CpG islands and histone modifications by methylation or acetylation of lysine residues. NATs and, more widely, ncRNAs, are thought to affect DNA methylation by interacting with various types of proteins involved in histone modification or chromatin remodeling such as, in particular, the polycomb repressive complex 2 (PRC2) [60]. A current hypothesis considers that nascent NATs guide PRC2 to specific-target sites on the chromatin. The tethering would occur by pairing the nascent NAT with DNA or mRNA sequences, during or after NAT transcription (Figure 4A) [61]. 

Additionally, a “decoy” mechanism can be described, where the NAT binds a protein complex, such as PRC2, and prevents this complex from binding the sense transcript by competition. This complex can also prevent the interaction of the sense gene with RNA Pol II or the chromatin [61,62].

Here are two examples of lncRNA/NAT that play a role in the tethering of PRC2 with chromatin. The first example is the combined action of ANRIL and PRC1–PCR2 on *INK4b-ARF-INK4a* gene expression and on the chromatin structure of this locus. ANRIL is a cisNAT that is dysregulated in breast cancer. It is located in the *INK4b-ARF-INK4a* gene cluster, which contains three genes encoding the three tumor-suppressor proteins p15, p14 and p16 [63]. Polycomb repressive complexes **1** and **2** (PRC1 and PRC2) are implicated in epigenetic silencing mechanisms. ANRIL can recruit those complexes to the chromatin of the *INK4b-ARF-INK4a* locus, recruiting PRC2 through interaction with SUZ12 and EZH2 components, and recruiting PRC1 by binding CBX7 [63,64,65]. Next, PRC2 silences *INK4b-ARF-INK4a* gene expression by inducing *H3K27* tri-methylation, and PRC1 maintains a repressive chromatin structure by mono-ubiquitination of *H2AK119* (Figure 4B) [66]. 

A second example is HOTAIR, which is implicated and dysregulated in many types of cancer and displays an active and critical role in chromatin dynamics [67,68]. Like ANRIL, HOTAIR interacts with PRC2 through its 5′ end to induce H3K27 tri-methylation. In addition, HOTAIR binds to LSD1 (lysine-specific demethylase 1) by its 3′ end, leading to H3K4 demethylation. These combined modifications lead, in trans, to a repressive chromatin structure and thus to the silencing of multiple genes [68,69]. 

#### 4.3.1. Double-Stranded RNA/RNA Masking

NATs can regulate gene expression through the formation of a complex of two overlapping NAT sequences. This double-stranded RNA (dsRNA) molecule thus creates a physical protection against post-transcriptional regulation factors that target the *pcGene*. RNA masking will then interfere with splicing or translation machineries. This mechanism will also prevent miRNA binding or RNAse activities, which often target single-stranded RNA and influence their complex stability [52]. Under this condition and in opposition with other mechanisms described above, NAT positively regulates *pcGene* expression. 

In osteocarcinoma, upregulated FGFR3-AS1 forms a tail-to-tail dsRNA with FGFR3, its sense transcript. FGFR3 mRNA is thus protected against RNase activity, leading to an increase in both its mRNA stability and its protein production [70]. Conversely, binding of the MALAT1 3′ UTR by its ncNAT TALAM1 allows for RNase P cleavage, leading to 3′ end processing and maturation that is essential for MALAT1 stability and function [71]. 

While forming dsRNAs, NATs can also interfere with splicing and translation mechanisms. For example, the protein coded from the gene *ZEB2* is a transcriptional factor that downregulates E-cadherin and its antisense transcript, ZEB2-AS1. *ZEB2* also contains an IRES (internal ribosome entry site) required for its translation. By binding this sequence, ZEB2-AS1 promotes ZEB2 splicing and downregulates its protein expression [72].

#### 4.3.2. Double-Stranded RNA/RNA A to I Editing

ADARs (adenosine deaminases that act on RNA) are enzymes responsible for RNA editing by site-specific adenosine deamination. They target dsRNA molecules such as those formed by NAT pairs. After adenosine to inosine (A-to-I) editing, inosines (I) are interpreted as a guanosines (G) during splicing or translation. Such modification may modulate the localization or the stability of the edited transcripts [73,74]. The occurrence frequency of RNA editing by NATs is not yet characterized [52,75,76]. Indeed, few NATs display edited sequences, but they may be quickly degraded or retained in the nucleus, thus disappearing from the bulk of the expressed sequences [77].

An example of this A to I editing mechanism has been found in human prostate cancers with the sense/antisense couple of PRUNE2 and PCA3 transcripts. *PRUNE2* is a *pcGene* that has a tumor suppressor role. PCA3 is an NAT that originates from introns, and is fully overlapped by *PRUNE2*’s 6th intron. The dsRNA created by PCA3 and PRUNE2’s pre-mRNA forms a complex with ADAR proteins. An A-to-I editing of this dsRNA leads to a downregulation of protein expression and an increase in tumor cell growth [78]. It is important to note that PCA3 was also approved as a specific biomarker for diagnostic tests.

#### 4.3.3. Double-Stranded RNA/RNA Interference

RNA interference is an additional mechanism whereby NATs are implicated in pcGene post-transcriptional regulation [79]. RNA interference is the endogenous siRNA formation from NAT-derived dsRNA. RNA interference is DICER-dependent and is followed by the action of the RNA-induced silencing complex (RISC) [80,81,82]. NATs may thus serve as precursors in endo-siRNA and miRNA production [83]. NATs form internal hairpins or duplexes with sense RNA, leading to a dsRNA that can be handled and digested by DICER. Short RNA duplexes will then be bound by the RISC complex, where one strand of the RNA duplex is used as a guide for mRNA recognition. This mRNA is then cleaved by the RISC complex, which will decrease the protein expression. Even with scarce evidence of NAT involvement in the RNA interference process, recent transcriptome sequencing studies have shown the widespread occurrence of endo-siRNAs and their regulatory potential during stages of development and differentiation [82,83]. 

## 5. NATs in Breast Cancer

Numerous studies have highlighted a link between lncRNA/NAT and cancers, especially breast cancers. Most of these transcripts were either highlighted by high-throughput transcriptomic studies that lacked the strand origin, or explored one by one due to their implication in oncogenic pathways. Therefore, many lncRNA listed in Table 1 are generally not described as NAT in the literature. In addition, the expression correlation between the NAT pair transcripts, as well as the ncNAT regulatory role with regard to the paired *pcGene*, are often unknown. It is also worth noting that most genomic loci coding for NAT transcript pairs also display numerous alternative transcripts. Therefore, each lncRNA transcript may belong to different classes among NAT pc|nc, lincRNA, lncRNA, or NAT nc|nc. 

To the best of our knowledge, only three strand-specific whole genome transcriptomic studies were performed on breast cancer samples [39,46,50]. The main concordant conclusions were that: (i) *pcGene* transcription coincides with an antisense ncNAT transcription in 50% of the cases; (ii) NAT transcripts are 1000 times less abundant than *pcGene* transcripts; and (iii) positive expression correlations between ncNATs and their paired *pcGenes* are approximately six times more frequent than negative correlations. This latest suggests that if ncNATs can affect the expression of their corresponding *pcGene*, positive regulation of expression should be more frequently observed than repression. However, a comparison of transcript levels between tumors and paired non-malignant adjacent healthy tissues showed that the ncNAT/*pcGene* transcript balance is disrupted in tumors. Therefore, new positive correlations of NAT/*pcGene* pairs are created in tumor tissues, while others that were present in the normal tissue decline [50].

The mechanism by which lncRNA/NAT regulates *pcGene* expression is known in several instances, and two mechanisms are often described in breast cancer. The first is driven by the polycomb repressing complexes (PRC), and the second by microRNAs. Here are three examples of PRC2 involvement in cancer pathways. The NAT ANRASSF1 leads PRC2 binding on the *RASSF1* promoter to regulate *RASSF1* expression [84]. The *INK4b-ARF-INK4A* locus coding for the cell cycle associated proteins p14, p15 and p16 is regulated by the NAT ANRIL via PRC2, and in addition, the lncRNA PANDAR recruits PRC1 to also regulate p16 expression [63,64,65,85]. Similarly, the p53 pathway is regulated at several levels via PCR2 by HOTAIR and MEG3 lncRNAs [86,87,88,89]. The importance of gene regulation by PRC2 is well known in breast cancers, as the expression of its targeted genes can be used to predict patient outcomes [90]. 

As displayed in Table 1, microRNAs are also frequently involved in gene regulation by lncRNA. One particular example is the epithelial to mesenchymal transition (EMT) that is regulated by three lncRNAs, namely, H19, linc-RoR and TP73-AS, which capture multiple microRNAs and prevent their binding to other mRNA targets [91,92,93,94].

### 5.1. NATs as Cancer Biomarkers

Like mRNAs, the expression levels of NATs and lncRNAs are affected under cancerous conditions. Differences in mRNA expression patterns between different subgroups of breast cancer patients have been used to develop genomic tests able to predict patient’s prognosis, or to predict treatment response by breast cancers. Among them, we can underline the MammaPrint and PAM50 microarray-based gene signatures, or the Oncotype DX RT-PCR–based assay that can help clinicians make treatment decisions based on the calculation of the recurrence risk, and/or the benefits of chemotherapy in the case of Oncotype DX test [179,180,181]. 

Similarly, multiple NATs/lncRNAs display expression levels that are associated with the disease prognosis, the treatment response or the clinical classification of breast cancers (Table 1). Although no clinically validated test has emerged yet, several studies report prognostic ncRNA gene signatures [119,182,183,184,185].

### 5.2. NATs as Therapeutic Targets

The understanding of antisense transcription is important for therapies. Indeed, NATs represent a potential highly specific entry point for therapeutic intervention on targeted genes by the use of ASO (antisense oligonucleotides) that are drugs already FDA-approved for several diseases [186]. 

Functionally characterized NATs can be targeted by ASOs, called in this case antagoNATs, to block the interaction of the sense and antisense transcripts. The hybridization of ASOs with the antisense transcript would lead to its degradation, or to transcriptional de-repression at the chromatin level [187]. The first in vivo demonstration of antagoNAT efficacy was shown by Modarresi et al. [188] and has been validated in other clinical contexts, detailed in the review by MacLeod et al. [187].

## 6. Conclusions

Up to 90% of the human genome length is transcribed: ~2% of the genomic DNA is coding for proteins; ~88% is transcribed but do not encode proteins; and ~10% is not transcribed. In contrast, ~80% of the RNA transcripts are coding for proteins and the remaining ~20% do not. These sequences are thus less expressed than the coding ones. They are also less conserved between species than coding genes, but more conserved than the non-coding and the non-transcribed genes. Such transcripts must therefore play a biological role, which has yet to be described.

Among lncRNAs, NATs are coding or non-coding RNA sequences, which are complementary to and overlapping with either protein-coding or non-coding transcripts. Their main biological role is thought to be the regulation of *pcGene* expression through a variety of molecular mechanisms. High-throughput transcriptomic studies have demonstrated that the expression of NATs and lncRNAs is modified under cancerous conditions, making them good cancer biomarkers. Finally, non-coding/*pcGene* transcript pairs are interesting, especially for specific target-gene treatments using ASO.

## Figures and Tables

**Table 1:** Role and therapeutic utility of lncRNAs in breast cancer. lncRNAs implicated in breast cancer pathology are listed and classified in different categories: lincRNA (long intergenic non-coding RNA), bidirectional lncRNA, sense-overlapping lncRNA, sense-intronic lncRNA, and NAT composed of nc|nc or nc|pc transcripts pairs. In the case of nc|pc pairs, the pcGene name is provided. As ncRNAs often display multiple transcript variants, some lncRNAs may belong to multiple categories.

lncRNA in Breast Cancer	Access Number	Type	*pcGene*	Alteration/Role in Breast Cancer	Mechanism of Action	Therapeutic Utility	Ref.
ANRASSF1	ENSG00000281358	NAT (nc|pc)	*RASSF1*	Upregulation/Oncogenic	Binds to PRC2 and silences the tumor suppressor gene RASSF1A.		[84]
ANRIL	ENSG00000240498	NAT (nc|pc)	*CDKN2A, CDKN2B*	Upregulation/Oncogenic	ANRIL is the NAT of CDKN2B gene (p15); binds to components of PRC1 (CBX7) and PRC2 (SUZ12) to silence the INK4 locus by epigenetic mechanisms.	Overexpressed in a variety of cancers and diseases.	[63,64,65]
LINC00901	ENSG00000242385	NAT (nc|pc)	*LSAMP*	Downregulation/Tumor suppressor	Low expression is associated with low overall survival.	Potential prognostic marker.	[95]
BCAR4	ENSG00000262117	lincRNA		Upregulation/Oncogenic	Interaction with SNIP1 and PNUTS in Hedgehog canonical pathway leads to a resistance to cancer treatments with SMO inhibitors.	Responsible for the acquisition of resistance to treatments and upregulation of non-canonical hedgehog pathway.	[96,97]
BCYRN1	ENSG00000236824	NAT (nc|nc)		Upregulation/Oncogenic	BCYRN1 expression is associated with cell proliferation.	Knockdown BCYRN1 impacts viability of actively proliferating cells through growth arrest and apoptosis. Potential therapeutic target for various cancers.	[98]
CCAT2	ENSG00000280997	NAT (nc|nc)		Upregulation/Oncogenic	Downregulates p15 through interaction with EZH2. Regulates TGF-β and Wnt signaling pathways. Promotes cell proliferation, invasion, tumor growth and metastasis.	Potential prognosis biomarker and therapeutic target.	[99,100,101,102,103]
CRNDE	ENSG00000245694	lincRNA		Upregulation/Oncogenic	Molecular sponge of miRNA-136 in breast cancer, activating Wnt/β-catenin.	Associated with unfavorable prognosis.	[104]
DANCR	ENSG00000226950	lincRNA		Upregulation/Oncogenic	Participates in cell proliferation and invasion.	Associated with a worse prognosis in TNBC.	[105]
DSCAM-AS1	ENSG00000235123	NAT (nc|pc)	*DSCAM*	Upregulation/Oncogenic	Expression induced by estrogen stimulation. Positive correlation with genes associated with cancer aggression, tamoxifen resistance, and metastasis.	Biomarker for luminal subtype.	[106,107]
FAM83H-AS1	ENSG00000282685	lincRNA		Upregulation/Oncogenic	Most upregulated in luminal subtype of breast cancer.	Prognostic marker of luminal subtype.	[108]
GAS5	ENSG00000234741	NAT (nc|pc)/bidirectional lncRNA/lincRNA (*multiple transcripts*)	*ZBTB37*	Downregulation/Tumor suppressor	Required for decoy of glucocorticoid receptor (GR), inhibits transcriptional induction by GR, stops growth and triggers apoptosis, induces PTEN through miR-103 inhibition.	Responsible for the acquisition of trastuzumab resistance. Potential circulating biomarker.	[109,110,111,112,113,114]
H19	ENSG00000130600	lincRNA		Upregulation/Oncogenic	Mediates breast cancer cell plasticity, invasion, and proliferation by sponging several miR (miR-200b/c, let-7b, miR-152), silences pro-apoptotic gene *BIK* through epigenetic modifications, precursor of miR-675 (pro-tumoral and pro-metastatic).	Upregulated in cancer. Potential circulating biomarker for early screening and prognosis monitoring in breast cancer.	[92,115,116,117,118]
HIF1A-AS2	ENSG00000258667	NAT (nc|pc)	*HIF1A*	Upregulation/Oncogenic	Involved in cell proliferation and invasion, contributes to chemotherapy resistance.	In TNBC, biomarker for detection, prognosis and prediction for recurrence and response to taxane chemotherapy.	[119,120]
HMMR-AS1	ENSG00000251018	NAT (nc|pc)	*HMMR*	Upregulation/Oncogenic	Involved in cell proliferation and invasion.	Positive correlation with HMMR, BRCA1, BRCA2 (oncogenic), biomarker and potential target in basal-like breast cancer.	[121]
HOTAIR	ENSG00000228630	NAT (nc|pc)/lincRNA (*multiple transcripts*)	*HOXC11*	Upregulation/Oncogenic	Guides epigenetic mechanisms to silence tumor suppressor genes through interaction with PRC2 and LSD1, involved in protein degradation by interaction with E3-ubiquitin ligases, tumor invasion, apoptosis and EMT.	Over-expressed in cancer, biomarker and potential therapeutic target.	[86,89,122,123,124,125,126,127]
HOTAIRM1	ENSG00000233429	NAT (nc|pc)/bidirectional lncRNA (*multiple transcripts*)	*HOXA1, HOXA2*	Upregulation/Oncogenic	Modulates gene expression in *HoxA* gene cluster by interacting with PRC1 and PRC2 complexes. High positive correlation with HOXA1 expression.	Increased in basal-like subtype breast cancer.	[128,129]
HOXA-AS2	ENSG00000253552	NAT (nc|pc)	*HOXA3, HOXA4*	Upregulation/Oncogenic	Acts as an endogenous sponge of miR-520c-3p and indirectly controls the expression of miR520c-3p target genes (*TGFBR2* and *RELA*).		[130]
HOXA11-AS	ENSG00000240990	NAT (nc|pc)	*HOXA11*	Upregulation/Oncogenic	Promotes cell proliferation, invasion and metastasis by regulating EMT.	Biomarker for metastasis and prognosis in breast cancer. Blocked relation between HOXA11-AS and EMT may have therapeutic utility.	[131]
Lnc-ITGB1-6:7	Lnc-ITGB1-6 (LNCipedia)	lincRNA		Upregulation/Oncogenic	Promotes cell proliferation, invasion and metastasis by regulating EMT. High linc-ITGB1 expression is associated with poor prognosis.	Biomarker in prognosis of breast cancer.	[132,133]
Linc-RoR	ENSG00000258609	lincRNA		Upregulation/Oncogenic	Induces EMT. Contributes to tumor growth, invasion, metastasis and drug resistance through endogenous competition with various miR (145, 205, 133, 34) and inhibition of p53 expression.	Upregulation is a marker in multi-drug resistance, chemotherapy tolerance. Potential therapeutic target for aggressive and metastatic breast cancer.	[91,134]
LINC00472	ENSG00000233237	lincRNA		Downregulation/Tumor suppressor	Associated with tumor grade, estrogen receptor status and molecular subtype in breast cancer. Repressed by methylation of its promoter.	Potential prognosis and predictive biomarker.	[135,136]
LSINCT5	ENSG00000281560	lincRNA		Upregulation/Oncogenic	Promotes cell proliferation.		[137]
MALAT1	ENSG00000251562	NAT (nc|nc)		Upregulation/Oncogenic	Plays a critical role in pre-mRNA alternative splicing. Regulates EMT gene expression.	Knockdown reduces cell growth, invasion, migration and differentiation into cystic tumors. Potential prognosis marker in ER− and prediction marker for endocrine treatment sensitivity in ER+.	[138,139,140,141]
MEG3	ENSG00000214548	NAT (nc|nc)		Downregulation/Tumor suppressor	Represses MDM2, leading to p53 accumulation. Silences genomic loci of TGFβ-associated genes by interaction with PRC2. Represses AKT signaling pathway. Inhibits EMT by sponging miR-421.	Expression promotes apoptosis, inhibits proliferation and angiogenesis.	[87,88,142,143]
MIR31HG (LOC554202)	ENSG00000171889	Sense-overlapping lncRNA		Downregulation/Tumor suppressor	Host gene of miR-31. Silenced in TNBC by promoter hypermethylation. Inhibits invasion-metastasis cascade by targeting pro-metastasis genes (i.e., RhoA and WAVE3).		[144,145,146]
NEAT1	ENSG00000245532	lincRNA		Upregulation/Oncogenic	Modulates miRNA biogenesis by organizing key components of paraspeckles and regulates transcription through protein sequestration into paraspeckles. Promotes proliferation and EMT. In ER+, NEAT1 is indispensable for interaction between FOXN3 and SINA3 complex. Regulates EZH2 through miR-101.	Overexpression of miR-548ar-3p downregulates NEAT1 and results in inhibition of cell growth.	[147,148,149,150,151,152]
PANDAR	ENSG00000281450	lincRNA		Upregulation/Oncogenic	Represses p16^INK4A^ expression through modulating the recruitment of Bmi1 to the p16^INK4A^ promoter. Removes cycle arrest possibility during G1/S transition.	Potential therapeutic target.	[85]
PTPRG-AS1	ENSG00000241472	NAT (nc|pc)	*PTPRG, C3ORF14*	Upregulation/Oncogenic		Differentially expressed between ER+ and ER− subtypes.	[153,154]
PVT1	ENSG00000249859	NAT (nc|pc)	*TMEM75*	Upregulation/Oncogenic	Co-operation between c-Myc and PVT1. Enhances c-Myc stability through inhibiting its phosphorylation.	Due to synergy between c-Myc and PVT1, silencing PVT1 expression decreases cell proliferation and increases apoptosis. Potential therapeutic target.	[155,156,157,158]
SNHG17	ENSG00000196756	lincRNA				Differentially expressed between ER+ and ER− subtypes. Low expression associated with overall survival. Expression correlates with tumor grade.	[154]
SOX2-OT	ENSG00000242808	NAT (nc|pc), sense-overlapping lncRNA, lincRNA	*DNAJC19*	Upregulation/Oncogenic	Through positive effect on SOX2 expression, SOX2OT plays a key role in pluripotency and tumorigenesis.	Potential prognosis marker and therapeutic target.	[159,160]
SPRY4-IT1	ENSG00000281881	Sense-intronic lncRNA		Upregulation/Oncogenic	Upregulates ZNF703 involved in the activation of the mTor signaling pathway. Promotes cell proliferation and inhibits apoptosis.	SPRY4-IT1 positively correlates with tumor size and pathological stage. Prognostic marker and potential therapeutic target.	[161,162]
TERRA (Telomeric repeat-containing RNA)		lncRNA		Misregulation	Transcribed from telomeric C-rich strand. Interacts with TRF1 and TRF2 to facilitate heterochromatin formation. Provides RNA template to aid telomerase function.	Potential therapeutic target to impair telomerase activity.	[163,164,165,166]
TP73-AS1	ENSG00000227372	NAT (nc|pc)	*TP73*	Upregulation/Oncogenic	TP73-AS1/miR-200a/ZEB1 forms a regulating loop. TP73-AS1 competes with ZEB1 for binding to miR-200a. ZEB1 binds to TP73-AS1 promoter and activates its expression. Upregulation of TP73-AS1/ZEB1 promotes cell invasion and migration.	Potential therapeutic target.	[94,167]
treRNA	ENSG00000231265	lincRNA		Upregulation/Oncogenic	Regulates translation through interaction with ribonucleoprotein complex, which will bind to the translation initiation factor (EIF4G1). Overexpressed in lymph-node metastasis. Promotes tumor invasion and metastasis. Regulates expression of metastasis promoting-gene *Snail*. Suppresses epithelial markers and translation of E-cadherin mRNA.		[168]
UCA1	ENSG00000214049	lincRNA		Upregulation/Oncogenic	Enhances chemotherapy resistance (tamoxifen) through mTor pathway inhibition and miR-18a downregulation. Promotes EMT through activating Wnt/β-catenin signaling. UCA1/hnRNP1 suppresses p27 protein level by competition. Downregulates tumor suppressor miR-143.	Potential urine biomarker. Knockdown reduces chemoresistance, cell migration and tumor size.	[169,170,171,172,173]
ZFAS1	ENSG00000177410	NAT (nc|pc)	*ZNFX1*	Downregulation in breast cancer/Upregulated in other cancers	Associated with ribosomes in breast cancer. Role in development and cell differentiation in mammary gland.	Potential biomarker.	[174,175,176,177,178]

Abbreviations: nc: non-coding; pc: protein coding; lncRNA: long intergenic non-coding RNA; TNBC: triple negative breast cancer; NAT: natural antisense transcript; EMT: epithelial to mesenchymal transition; ER+ and ER−: estrogen receptor positive and negative breast cancers; G1/S transition: The transition between the two first phases of the cell cycle; ANRASSF1: RASSF1 antisense RNA 1; RASSF1: Ras association domain family member 1; PRC: polycomb repressive complex; ANRIL: antisense non-coding RNA in the INK4 locus; CDKN2A: cyclin dependent kinase inhibitor 2A; CDKN2B: cyclin dependent kinase inhibitor 2B; CBX7: chromobox 7; SUZ12: SUZ12 polycomb repressive complex 2 subunit; INK: cyclin dependent kinase inhibitor; LSAMP: limbic system-associated membrane protein; BCAR4: breast cancer anti-estrogen resistance 4; SNIP: SRC kinase signaling inhibitor 1; PNUTS: protein phosphatase 1 regulatory subunit; SMO: smoothened, frizzled class receptor; BCYRN1: brain cytoplasmic RNA 1; CCAT2: colon cancer associated transcript 2; TGFbeta: transforming growth factor beta 1; Wnt: wingless-type MMTV integration site family; CRNDE: colorectal neoplasia differentially expressed; DANCR: differentiation antagonizing non-protein coding RNA; DSCAM-AS1: DSCAM antisense RNA 1; DSCAM: DS cell adhesion molecule; FAM83H-AS1: FAM83H antisense RNA 1; GAS5: growth arrest specific 5; ZBTB37: zinc finger and BTB domain containing 37; GR: glucocorticoïd receptor; H19: H19, imprinted maternally expressed transcript; BIK: BCL2 interacting killer; HIF1A-AS2: HIF1A antisense RNA 2; HIF1A: hypoxia inducible factor 1 alpha subunit; HMMR-AS1: HMMR antisense RNA 1; HMMR: hyaluronan mediated motility receptor; BRCA1: BRCA1, DNA repair associated; BRCA2: BRCA2, DNA repair associated; HOTAIR: HOX transcript antisense RNA; HOXC11: homeobox C11; LSD1: lysine demethylase 1A; HOTAIRM1: HOXA transcript antisense RNA, myeloid-specific 1; HOX: homeobox; HOXA-AS2: HOXA cluster antisense RNA 2; TGFBR2: transforming growth factor beta receptor 2; RELA: RELA proto-oncogene, NF-kB subunit; HOXA11-AS: HOXA11 antisense RNA; LSINCT5: long stress-induced non-coding transcript 5; MALAT1: metastasis associated lung adenocarcinoma transcript 1; MEG3: maternally expressed 3; MDM2: transformed mouse 3T3 cell double minute 2 proto-oncogene; AKT: thymoma viral proto-oncogene serine/threonine kinase 1; RhoA: ras homolog family member A; RhoA: ras homolog family member A; WAVE3: WAS protein family member 3; NEAT1: nuclear paraspeckle assembly transcript 1; FOXN3: forkhead box N3; SINA3: E3 ubiquitin-protein ligase SINAT5-like; PANDAR: promoter of CDKN1A antisense DNA damage activated RNA; Bmi1: BMI1 proto-oncogene, polycomb ring finger; PTPRG-AS1: PTPRG antisense RNA 1; PTPRG: protein tyrosine phosphatase, receptor type G; C3ORF14: chromosome 3 open reading frame 14; PVT1: Pvt1 oncogene; TMEM75: transmembrane protein 75; c-Myc: MYC proto-oncogene, bHLH transcription factor; SNHG17: small nucleolar RNA host gene 17; SOX2-OT: SOX2 overlapping transcript; DNAJC19: DnaJ heat shock protein family (Hsp40) member C19; SOX2: SRY-box 2; SPRY4-IT1: SPRY4 intronic transcript 1; ZNF703: zinc finger protein 703; mTor: mechanistic target of rapamycin kinase; TRF: telomeric repeat binding factor; TP73-AS1: TP73 antisense RNA 1; TP73: tumor protein p73; ZEB1: zinc finger E-box binding homeobox 1; treRNA: translation regulatory long non-coding RNA 1; EIF4G1: eukaryotic translation initiation factor 4 gamma 1; Snail: snail family transcriptional repressor 1; UCA1: urothelial cancer associated 1; ZFAS1: ZNFX1 antisense RNA 1; ZNFX1: zinc finger NFX1-type containing 1.

## Abbreviations

NAT	Natural antisense transcripts
nc	Non-coding
pc	Protein-coding
ds	Double-stranded
*pcGene*	Protein-coding gene
lncRNA	Long non-coding RNA
lincRNA	Long intergenic non-coding RNA
ncRNA	Non-coding RNA
mRNA	Messenger RNA
RNA Pol II	RNA polymerase II
PRC	Polycomb Repressive Complex
ASO	Antisense oligonucleotide
